# Clinicopathological Characteristics of Pleomorphic High-Grade Squamous Intraepithelial Lesion of the Uterine Cervix: A Single-Institutional Series of 31 Cases

**DOI:** 10.3390/diagnostics10080595

**Published:** 2020-08-15

**Authors:** Hyunjin Kim, Sangjoon Choi, Sung-Im Do, Sang Hwa Lee, Nara Yoon, Hyun-Soo Kim

**Affiliations:** 1 Department of Pathology and Translational Genomics, Samsung Medical Center, Sungkyunkwan University School of Medicine, Seoul 06351, Korea; h2407.kim@samsung.com (H.K.); choisj88@gmail.com (S.C.); 2 Department of Pathology, Kangbuk Samsung Hospital, Sungkyunkwan University School of Medicine, Seoul 03181, Korea; sungim.do@samsung.com; 3 Pathology Center, Seegene Medical Foundation, Seoul 04805, Korea; lsh@mf.seegene.com; 4 Department of Pathology, Incheon St. Mary’s Hospital, College of Medicine, The Catholic University of Korea, Incheon 21431, Korea

**Keywords:** cervix, high-grade squamous intraepithelial lesion, pleomorphic variant, Ki-67

## Abstract

We investigated the clinicopathological characteristics of 31 cases of pleomorphic high-grade squamous intraepithelial lesions (PHSIL) of the uterine cervix. We reviewed electronic medical records and all available slides to collect clinical and pathological information. PHSILs were histologically characterized by significant nuclear enlargement, marked pleomorphism, hyperchromasia, increased mitotic activity, and frequent atypical mitoses. In the majority of cases (24/31; 77.4%), this striking nuclear atypia involved both the surface epithelium and the endocervical glands. In the remaining seven cases, pleomorphic cells were observed in the surface epithelium only. PHSILs involving both the surface epithelium and glands showed higher mitotic counts and Ki-67 labelling indices than the surface-only PHSILs. Invasive squamous cell carcinoma was present in only one case (3.2%), and none developed recurrent disease. Our observations of striking nuclear atypia in cases of HSIL did not indicate increased aggressiveness. Further investigations are required for confirmation of our data in larger cohorts.

## 1. Introduction

Cervical cancer is the fourth most common malignancy in women and remains the leading cause of cancer-related death in resource-poor countries [1]. The Lower Anogenital Squamous Terminology Standardization Project [2] and the World Health Organization Classification of Tumors of Female Reproductive Organs [3] advise that cervical intraepithelial lesions be diagnosed as either low- or high-grade to improve the simplicity, accuracy, and reproducibility of diagnosis for these precancerous lesions. Low-grade squamous intraepithelial lesions (LSILs) have a low risk of recurrence or of developing into cancer. In contrast, high-grade squamous intraepithelial lesions (HSILs) of the uterine cervix are premalignant lesions that are associated with a significant risk of invasive squamous cell carcinoma [3]. Ten percent of HSIL cases have been shown to progress to invasive carcinoma within two to ten years [4]. Therefore, HSILs should be treated properly as they may be a warning sign for invasive carcinoma.

The characteristic histological features of HSILs are familiar to all surgical pathologists who examine cervical biopsies or hysterectomy specimens [5]. The pathological diagnosis of a HSIL is straightforward in the great majority of cases, although potential histological mimics such as atrophy, transitional metaplasia, radiation effects, and reactive changes occasionally present difficulties [5]. We have recently experienced several cases of HSIL that displayed striking focal or diffuse nuclear enlargement and pleomorphism, often accompanied by increased mitotic activity and frequent atypical mitoses. Although these atypical nuclear features are very unusual, they are readily recognizable at scanning magnification. The nature and significance of these nuclear alterations in HSILs have previously been reported in only a single study [5], in which this rare variant of HSIL was referred to as a pleomorphic HSIL (PHSIL). In this study, we investigated the clinicopathological characteristics and immunostaining results of a single-institutional series of 31 cases of PHSIL.

## 2. Materials and Methods

### 2.1. Case Selection

This study was reviewed and approved by the Institutional Review Board (permission code: 2020-07-049-001; approved on 9 July 2020). All participants provided written informed consent. During the one-year study period from May 2019 to April 2020, 31 patients were diagnosed as having a PHSIL. Clinical information, including age at diagnosis, cytological diagnosis, human papillomavirus (HPV) status, histological diagnosis, and recurrence was obtained from the electrical medical information systems and pathology reports. The cytological diagnoses were established according to the Bethesda System for Reporting Cervical Cytology [6] and were classified as follows: squamous cell carcinoma (SCC), HSIL, atypical squamous cells, cannot exclude HSIL (ASC-H), LSIL, atypical squamous of undetermined significance (ASC-US), and negative for intraepithelial lesion or malignancy (NILM). The histological diagnoses of SCC and HSIL were made according to the morphological criteria recommended by the Lower Anogenital Squamous Terminology Standardization Project [2] and the World Health Organization Classification of Tumors of Female Reproductive Organs [3].

### 2.2. Pathological Examination

PHSIL was defined as HSIL exhibiting striking nuclear atypia, including marked pleomorphism, hyperchromasia [5], and significant nuclear enlargement (more than four times larger than normal basal cells), which was easily identifiable at scanning magnification. All hematoxylin and eosin-stained slides were thoroughly examined by a board-certified pathologist specialized in gynecological oncology (H.-S.K.). Pathological information, including the largest dimension of each PHSIL, the location of pleomorphic cells, and the number of mitotic figures (including atypical ones), was analyzed. The most representative slide was chosen from each case to perform immunohistochemical staining for p16 and Ki-67. In addition, we also compared the frequencies of coexisting SCC and disease recurrence in PHSIL cases with those of a separate consecutive series of 124 conventional HSILs diagnosed in conization specimens.

### 2.3. Immunohistochemical Staining

Four-micrometer-thick slides were cut from formalin-fixed, paraffin-embedded tissue sections. The slices were then deparaffinized and rehydrated with a xylene and alcohol solution. Immunostaining was performed using an automatic instrument [7,8,9,10,11,12,13,14,15]. After antigen retrieval, the slices were incubated with primary antibodies including p16 (prediluted, clone E6H4, Ventana Medical Systems, Oro Valley, AZ, USA) and Ki-67 (1:200, clone MIB-1, Dako, Glostrup, Denmark). After chromogenic visualization, the slices were counterstained with hematoxylin. Appropriate positive and negative controls were concurrently stained to validate the staining method. Endometrial serous carcinoma and ovarian high-grade serous carcinoma, both of which exhibit diffuse and strong nuclear p16 immunoreactivities and very high (>90%) Ki-67 labeling indices, were used as positive controls. The negative control was prepared by substituting non-immune serum for primary antibody and resulted in no detectable staining. The p16 immunostaining pattern was interpreted as block positive when p16 expression was strong and continuous along the basal and parabasal layers, and involved nuclear or nuclear plus cytoplasmic staining. All other p16 immunostaining patterns, described as focal nuclear staining or wispy, blob-like, puddled, or scattered cytoplasmic staining, were interpreted as patchy positive [2,11,14,15,16,17,18,19,20,21,22]. 

## 3. Results

### 3.1. Clinical Characteristics

The clinicopathological characteristics are summarized in Table 1. The patients’ ages ranged from 25 to 80 (median = 44; mean = 49.2) years. On cytological examination, two (6.5%) patients were diagnosed as having SCC, while 13 (41.9%) patients were diagnosed with HSIL, and seven (22.6%) patients with ASC-H. The combined cytohistological concordance rate was 71.0% (22/31). Three (9.7%) patients were diagnosed cytologically as having LSIL, one (3.2%) patient with ASC-US, and five (16.1%) patients with NILM. Information about HPV infection status was available for 23 patients, 21 (91.3%) of whom were infected. Coinfection with more than two HPV types occurred in 6 (26.1%) of the 23 cases. The infected HPV was not predominated by one specific type; the most common types were 16 and 51 (5/21, 23.8%; both), followed by 35 and 56 (3/21, 14.3%; both).

All patients underwent cervical punch biopsy. Twenty-four (77.4%) patients underwent conization, and three of these patients underwent subsequent hysterectomy. Of the seven patients who did not receive conization, three underwent hysterectomy. The remaining four patients did not receive further surgical procedures after punch biopsy.

Information about follow-up cytological examinations was available for nine (29.0%) patients, ranging from seven to nine months after the surgical procedures. All cases were diagnosed as NILM. Twenty-one (67.7%) cases were recent (< 6 months), and one (3.2%) patient was lost to follow-up. Among the comparative series of 124 conventional HSILs, two (1.6%) showed disease recurrence.

### 3.2. Pathological Characteristics

Punch biopsy revealed PHSIL in 29 (93.5%) patients, while the histological diagnoses of the remaining two (6.5%) patients were atypical cells and LSIL, respectively. A diagnosis of cervical intraepithelial neoplasia (CIN) 2 was assigned to five (16.1%) cases, while CIN 3 was assigned to 24 (77.4%) cases. In addition, the punch biopsy specimens of seven cases were interpreted as at least PHSIL (CIN 3), where SCC could not be excluded. The presence of PHSILs was confirmed in 87.5% (21/24) of the patients who underwent conization. The remaining three patients did not have any residual PHSIL in their conization specimen. Five of the six hysterectomy specimens had residual PHSIL.

**Figure 1:**
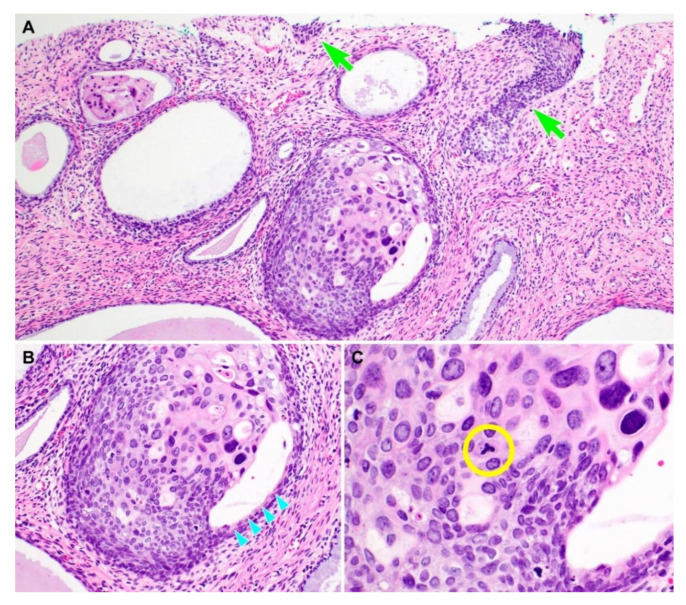
Histological features of pleomorphic high-grade squamous intraepithelial lesion (PHSIL). (**A**) Compared to conventional HSIL (two light green arrows), PHSIL (middle lower) displays significant nuclear enlargement and hyperchromasia that is easily identifiable at low-power magnification. (**B**) PHSIL exhibits expansile involvement of the endocervical gland. The presence of residual endocervical glandular epithelium along the luminal surface (four light blue arrowheads) indicates the intraepithelial nature. (**C**) An abnormal mitotic figure is detected (yellow circle). Hematoxylin and eosin staining. Original magnification: (**A**) = 100×, (**B**) = 200×, (**C**) = 400×.

**Figure 2:**
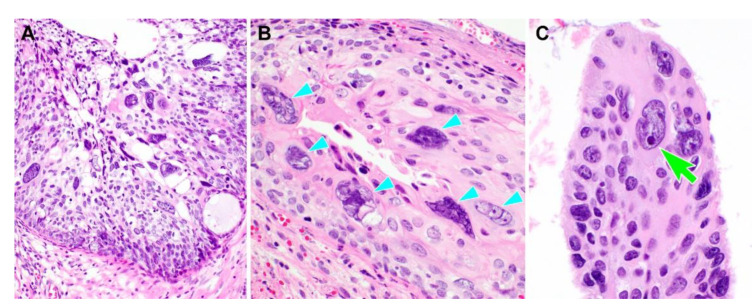
Nuclear features of pleomorphic high-grade squamous intraepithelial lesion (PHSIL). (**A**) Several scattered pleomorphic cells possess large, pleomorphic nuclei and variable amounts of clear or eosinophilic cytoplasm. A small, round residual endocervical lumen is noted (right lower corner). (**B,C**) PHSIL frequently displays (**B**) multinucleation (six light blue arrowheads) and (**C**) conspicuous nucleoli (a light green arrow). Hematoxylin and eosin staining. Original magnification: (**A**) = 200×, (**B**) = 400×, (**C**) = 600×.

**Figure 3:**
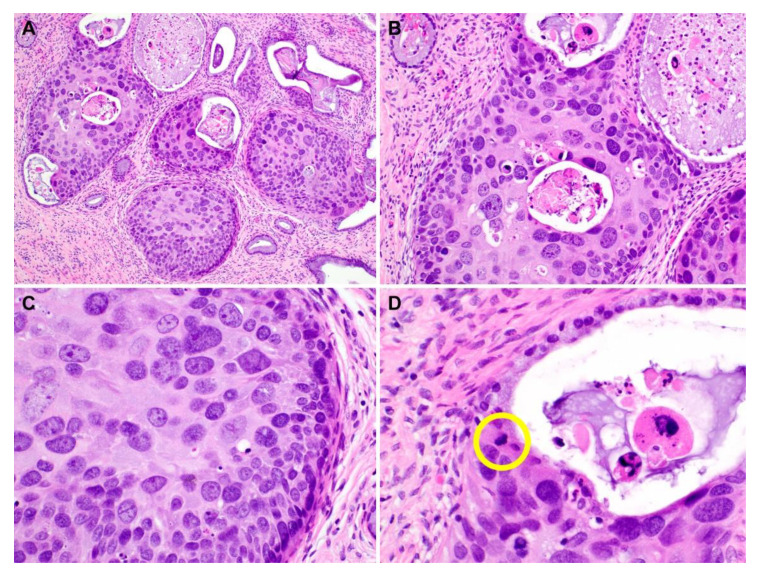
Histological features of pleomorphic high-grade squamous intraepithelial lesion (PHSIL) involving the endocervical glands. (**A**) PHSIL involves multiple endocervical glands. (**B**) The diameter of pleomorphic nuclei is more than five times larger than the basal cells and the adjacent normal endocervical glandular epithelium. (**C**) The nuclei display severe pleomorphism and hyperchromasia. Degenerative-appearing chromatin pattern is not evident. (**D**) An admixture of mucin and necrotic and parakeratotic cellular debris is noted within the lumen. A mitotic figure is detected (a yellow circle). Hematoxylin and eosin staining. Original magnification: (**A**) = 100×, (**B**) = 200×, (**C,D**) = 600×.

Representative photomicrographs showing histological features of PHSILs are shown in Figure 1. The diameter of PHSIL nuclei was at least four times larger than that of normal basal cells. Enlarged endocervical glands due to PHSIL involvement were readily identifiable at scanning magnification. Markedly increased mitotic activity and frequent atypical mitotic figures were observed. There were obvious nuclear pleomorphism and hyperchromasia as well as large, conspicuous nucleoli and frequent multinucleation (Figure 2). PHSILs involved both the surface epithelium and endocervical glands in 24 (77.4%) cases and the surface epithelium alone in seven (22.6%) cases. All PHSIL cases with endocervical glandular extension showed expansive growth. In all but two cases involving the glands, PHSILs involved multiple endocervical glands (Figure 3). The presence of variable-sized endocervical lumina at the periphery of the involved glands indicated their intraepithelial nature. Intraluminal necrotic and parakeratotic cellular debris with admixed mucin was frequently observed.

**Figure 4:**
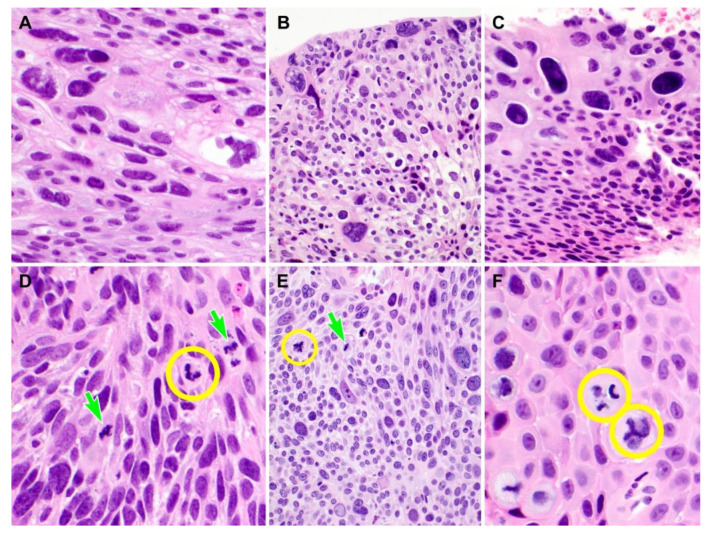
Difference in mitotic activity according to the microanatomical distribution of pleomorphic high-grade squamous intraepithelial lesion (PHSIL). (**A**–**C**) PHSILs involving the surface epithelium only show minimal or no mitotic activity. (**D**–**F**) PHSILs involving the endocervical glands show frequent typical mitoses (three light green arrows). Atypical mitotic figures (four yellow circles), such as asymmetric and tripolar mitoses, were noted. Hematoxylin and eosin staining. Original magnification: (**A**) = 400×, (**B**) = 200×, (**C**) = 400×, (**D**) = 600×, (**E**) = 200×, (**F**) = 600×.

We counted the number of typical and atypical mitoses to determine whether the mitotic count was different between PHSIL cases involving both the surface and glands, and those involving the surface only (Figure 4). The mean number of typical mitotic figures in the group involving both the surface and the glands (8.6; range = 1–24) was approximately two times greater than that of the group involving the surface only (4.1; range = 2–7). Furthermore, of the 24 PHSIL cases involving both the surface and the glands, all but two (91.7%) had at least one atypical mitotic figure (Figure 4). The highest count of atypical mitoses was eight (median = 2).

**Figure 5:**
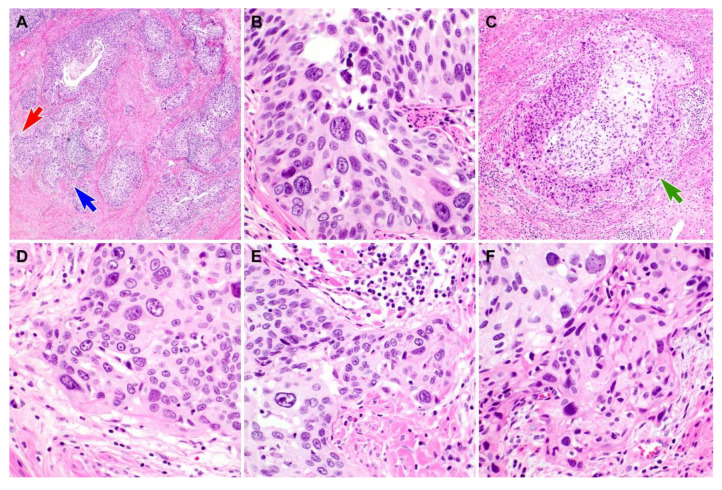
Histological features of squamous cell carcinoma associated with pleomorphic high-grade squamous intraepithelial lesion (PHSIL). (**A**) PHSIL extensively involves multiple endocervical glands. (**B**) PHSIL involving the endocervical glands displays marked nuclear enlargement and pleomorphism and red conspicuous nucleoli. (**C**) The largest invasive focus measures <1 mm. (**D**–**F**) High-power magnifications of microscopic invasive foci indicated by (**D**) red and (**E**) blue arrows in image (**A**) and by a (**F**) green arrow in image (**C**). Stromal desmoplasia and inflammatory infiltrates are associated. Hematoxylin and eosin staining. Original magnification: (**A**) = 40×, (**B**) = 400×, (**C**) = 100×, (**D**–**F**) = 200×.

Invasive SCC was identified in one (3.2%) patient, whose conization and hysterectomy specimens displayed both SCC and PHSILs (Figure 5). Some separate foci of stromal invasion were also detected. All invasive foci detected by microscopy were associated with PHSILs. The largest dimension measured <1 mm and the deepest invasion depth measured <1 mm (International Federation of Gynecology and Obstetrics (FIGO) stage IA1) [23]. Lymphovascular invasion was identified adjacent to the largest invasive focus. None of the 124 cases of conventional HSIL in the comparative series showed invasive SCC.

Information about the size of the PHSILs was available in 27 (87.1%) patients who underwent either conization, hysterectomy, or both procedures. The dimension of the largest PHSIL ranged from one to 18 (median = 5; mean = 5.5) mm. Three (11.1%) patients were found to have PHSILs measuring ≥10 mm (10 mm, 13 mm, and 18 mm, respectively). In contrast, seven (25.9%) patients had a PHSIL of ≤2 mm. In two of these seven patients, the size of the PHSIL in punch biopsy specimens measured ≤1 mm. The exocervical margin of the conization specimen showed PHSIL involvement in three patients, but none of these underwent hysterectomy.

**Figure 6:**
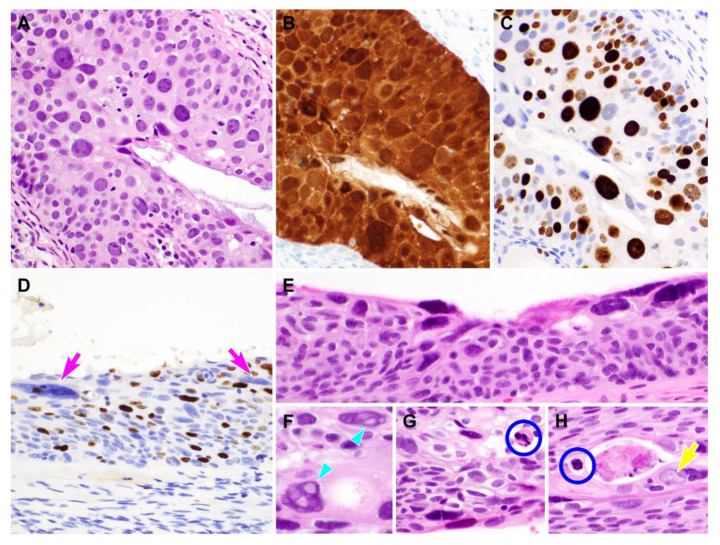
Immunostaining results of pleomorphic high-grade squamous intraepithelial lesion (PHSIL). (**A–C**) PHSIL involving the endocervical glands demonstrates (**B**) block p16 positivity and (**C**) strong Ki-67 expression in most of the pleomorphic cells. (**D–H**) PHSIL cells (**D**: two pink arrows) involving the surface epithelium only are not highlighted by Ki-67. (**E**–**H**) In hematoxylin and eosin-stained slides, (**E**) Ki-67-negative nuclei are mainly located along the luminal surface. (**F**) They show degenerative-appearing chromatin patterns, such as (**F**) intranuclear vacuoles (two light blue arrowheads), (**G,H**) pyknosis (two blue circles), and (**H**) ground glass appearance due to the intranuclear inclusion filling the entire nucleus (a yellow arrow). Hematoxylin and eosin staining: (**A**), (**E**), (**F–H**), polymer method: (**B–D**). Original magnification: (**A–H**) = 200×.

All (31/31; 100.0%) cases of PHSILs showed strong and diffuse nuclear and cytoplasmic p16 immunoreactivities (block p16 positivity; Figure 6). There was a difference in Ki-67 labelling indices between PHSIL cases involving both the surface and glands, and those involving only the surface. In 18 of the 24 (75.0%) cases of PHSIL involving both locations, most of the pleomorphic cells were strongly labelled by Ki-67 (Figure 6). In contrast, in six of the seven (85.7%) surface epithelium-only PHSILs, the pleomorphic cells were not labelled by Ki-67. In these cases, the pleomorphic cells displayed chromatin patterns that were more degenerative in appearance, showing smudged chromatin, pyknosis, multiple intranuclear vacuoles, or ground glass nuclei (Figure 6).

## 4. Discussion

PHSILs have previously been described by Stewart as markedly enlarged atypical squamous cells observed in areas of HSILs [5]. Based on this histological description, we selected 31 cases of PHSIL. The findings in our case series were consistent with those of Stewart on several points. Firstly, the majority of our PHSIL cases (24) involved both the surface epithelium and the endocervical glands. Secondly, one case of invasive SCC in our series had only a few microscopic invasive foci (<1 mm) and was FIGO stage IA1, similar to three invasive cases described in the previous study. Thirdly, there was no case of recurrence detected by follow-up cytological examination in either study (19 cases in Stewart [5] and 31 in the present study). During our short follow-up period, the marked nuclear pleomorphism of PHSILs did not correlate with more aggressive behavior than that of conventional HSILs. These observations were in concordance with those of Stewart, while contrasting with the commonly held opinion that pleomorphic cells reflect an aggressive nature. Lastly, almost all of the cases in our study (91.3%) exhibited block positivity for p16, findings that were once again consistent with those previously described by Stewart. This confirmed the association of HPV with PHSIL as well as the conventional HSIL.

There were also findings from this case series that differed from those of Stewart. Firstly, the number of cases and the age distribution of patients were different. We collected 31 consecutive cases from a single institution within 12 months, and the mean and median age of patients was 49.2 and 44 years, respectively. In contrast, Stewart collected 19 cases over a 5-year period, including 13 cases from his institution and 6 cases from consultations. The mean and median ages were much younger than the patients in our study, at 33.2 and 28 years, respectively. Secondly, we observed that PHSIL cells showed strong expression of Ki-67, similar to that of conventional HSIL cells in approximately two-thirds (19/31) of cases, all except one of which involved both the surface epithelium and the endocervical glands. In contrast, Stewart reported that Ki-67 labelling indices were variable in PHSILs, and that there was less staining in PHSIL cells compared to those of conventional HSILs [5]. Examining six of our cases which showed pleomorphic cells without Ki-67 labelling, we found that the pleomorphic cells were only located in the surface epithelium and did not involve the glands. Comparing the pleomorphic cells with and without Ki-67 labelling, we found that those without Ki-67 expression were mainly located in the surface epithelium and showed a more degenerative pattern. This microanatomical difference in Ki-67 expression suggests that the nuclear enlargement and pleomorphism involving the endocervical glands reflect proliferative activities, whereas those at the surface epithelium are more likely to be associated with degenerative activities such as cellular senescence or growth arrest. Thirdly, in our study we identified one case of invasive SCC that co-occurred with the PHSIL; however, the three SCC cases observed in Stewart’s study were only associated with conventional HSILs, rather than PHSILs. Finally, Stewart stated that the mitotic figures were rarely seen in the pleomorphic cells regardless of distribution. However, we clearly observed that PHSILs involving glands showed an average of 8.6 mitoses, twice more than what we observed in surface-only PHSILs. Considering the higher mitotic counts, atypical mitoses, as well as increased Ki-67 expression, it is clear that pleomorphic tumor cells located in the endocervical glands have higher proliferative potential compared to those located at the surface. Although the use of the mitotic index as a predictor of invasion or recurrence in HSILs has not yet been established, a high mitotic rate is a general indicator of more aggressive tumors. Longer follow-up of patients with PHSIL is necessary to determine the clinical significance of endocervical gland involvement of pleomorphic cells.

Considering the results from Stewart with those of the present, we cannot conclude that PHSIL has a more aggressive biological behavior compared to that of conventional HSILs, since SCC is seldom accompanied by PHSILs and there was no case of recurrence. Rather, in the comparative case series of conventional HSILs, two of the 124 patients developed recurrent HSIL. Therefore, when pathologists encounter PHSIL in a small cervical biopsy or a resected specimen, they should not presume that the lesion is more likely to recur or coexist with SCC. 

Ondic and Alaghehbandan described the histological features of PHSIL as being similar to those of bizarre cell dysplasia (BCD) [24,25]. Although there is a morphological overlap between PHSIL and BCD, significant differences exist, particularly in regards to the distribution of the bizarre cells. Ondic and Alaghehbandan defined BCD as a subtype of HSIL characterized by the presence of individual bizarre cells scattered irregularly throughout the epithelial thickness [25]. They stated that the bizarre cells showed nucleomegaly, anisonucleosis, multinucleation with overlapping, and variable hyperchromasia; however, their definition of the nucleomegaly was not clear. In contrast, PHSIL cells are described as having nuclei at least four times larger than those of normal basal cells, with severe pleomorphism and consistent hyperchromasia. Moreover, only some of the BCD cases showed endocervical glandular extension, but the majority of PHSIL cases, both those described by Stewart (78.9%) and those observed in our study (77.4%), showed glandular involvement. Despite these differences, both PHSILs and BCD show an association with high-risk HPV infection, are found with conventional HSILs, and show similar nuclear morphology, suggesting that these might not be two separate entities. We agree with the authors of these previous studies that further investigation is required to clarify the significance of BCD and PHSILs, which would lead to improved patient management.

## 5. Conclusions

PHSILs do not by themselves indicate a worse biological outcome than those without pleomorphic cells. There is a tendency for pleomorphic cells confined to the surface epithelium to demonstrate degenerative change with no Ki-67 expression. However, our observations of increased mitotic activity, frequent atypical mitoses, and increased Ki-67 labelling indices in PHSILs involving the endocervical glands should be further investigated with a larger cohort for their clinicopathological significance.

## Figures and Tables

**Table 1:** Clinicopathological characteristics of 31 patients with pleomorphic high-grade squamous intraepithelial lesion (PHSIL).

Case No.	Age (Years)	Cytological Diagnosis	HPV Status (Type)	Histological Diagnosis			Greatest Dimension (mm)	Endocervical Glandular Involvement	Number of Typical Mitosis	Number of Atypical Mitosis
				Biopsy	Conization	Hysterectomy				
1	55	HSIL	HR (51)	atypical cells	PHSIL (CIN 3)	NA	2	Present	3	1
2	52	ASC-H	HR (35, 52, 56)	LSIL (CIN 1)	PHSIL (CIN 3)	NA	6	Absent	7	0
3	41	NILM	NA	PHSIL (CIN 2)	PHSIL (CIN 2)	NA	6	Absent	3	0
4	44	HSIL	HR (18, 35)	PHSIL (CIN 2)	PHSIL (CIN 3)	NA	4	Present	8	1
5	38	HSIL	HR (51)	PHSIL (CIN 2)	PHSIL (CIN 3)	NA	5	Present	4	2
6	48	LSIL	NA	PHSIL (CIN 2)	No residual PHSIL	NA	3	Present	5	1
7	37	ASC-US	HR (58)	PHSIL (CIN 2; 1 mm)	PHSIL (CIN 3)	NA	2	Present	5	1
8	69	HSIL	NA	PHSIL (CIN 3)	SCC; PHSIL (CIN 3)	SCC; PHSIL (CIN 3)	1 (SCC); 11 (HSIL)	Present	17	3
9	40	HSIL	HR (16, 58)	PHSIL (CIN 3)	PHSIL (CIN 3)	NA	8	Absent	4	0
10	42	NILM	NA	PHSIL (CIN 3)	PHSIL (CIN 3)	NA	10	Present	9	1
11	27	ASC-H	NA	PHSIL (CIN 3)	PHSIL (CIN 3)	NA	4	Present	14	6
12	43	HSIL	HR (33)	PHSIL (CIN 3)	PHSIL (CIN 3)	NA	18	Present	24	7
13	25	NILM	NA	PHSIL (CIN 3)	PHSIL (CIN 3)	NA	4	Absent	8	0
14	72	NILM	NA	PHSIL (CIN 3)	PHSIL (CIN 3)	NA	4	Present	4	1
15	49	NILM	HR (31)	PHSIL (CIN 3)	PHSIL (CIN 3)	NA	13	Present	14	3
16	44	LSIL	HR (51)	PHSIL (CIN 3)	PHSIL (CIN 3)	NA	4	Present	1	0
17	44	HSIL	Not detected	PHSIL (CIN 3)	PHSIL (CIN 3)	NA	2	Present	4	1
18	49	HSIL	HR (18)	PHSIL (CIN 3)	NA	PHSIL (CIN 3)	2	Present	8	1
19	55	HSIL	Not detected	PHSIL (CIN 3)	NA	PHSIL (CIN 3)	6	Present	8	2
20	33	ASC-H	HR (35, 53)	PHSIL (CIN 3)	NA	NA	NA	Present	12	3
21	41	HSIL	HR (16)	PHSIL (CIN 3)	NA	NA	NA	Present	9	2
22	36	SCC	HR (51)	PHSIL (CIN 3)	NA	NA	NA	Present	4	1
23	42	LSIL	HR (56)	PHSIL (CIN 3)	NA	NA	NA	Present	9	3
24	60	ASC-H	HR (69)	PHSIL (CIN 3; < 1 mm)	PHSIL (CIN 3)	NA	1	Absent	3	0
25	80	ASC-H	HR (53)	At least PHSIL (CIN 3)	PHSIL (CIN 3)	PHSIL (CIN 3)	6	Present	1	0
26	80	HSIL	NA	At least PHSIL (CIN 3)	PHSIL (CIN 3)	PHSIL (CIN 3)	6	Absent	2	0
27	34	ASC-H	HR (16, 33)	At least PHSIL (CIN 3)	PHSIL (CIN 3)	NA	5	Present	22	8
28	62	ASC-H	HR (16)	At least PHSIL (CIN 3)	PHSIL (CIN 3)	NA	2	Present	5	2
29	74	HSIL	HR (16, 56)	At least PHSIL (CIN 3)	No residual PHSIL	NA	5	Present	8	1
30	33	HSIL	HR (51)	At least PHSIL (CIN 3)	No residual PHSIL	NA	5	Present	9	3
31	77	SCC	HR (52)	At least PHSIL (CIN 3)	NA	No residual PHSIL	5	Absent	2	0

ASC-US: Atypical squamous cells of undetermined significance, ASC-H: atypical squamous cells, cannot exclude HSIL, CIN: cervical intraepithelial neoplasia, HPV: human papillomavirus, HSIL: high-grade squamous intraepithelial lesion, HR: high-risk, LSIL: low-grade squamous intraepithelial lesion, NA: not applicable, NILM: negative for intraepithelial lesion or malignancy, PHSIL: pleomorphic high-grade squamous intraepithelial lesion, SCC: squamous cell carcinoma.

## Abbreviations

LSIL	Low-grade squamous intraepithelial lesion
HSIL	High-grade squamous intraepithelial lesion
PHSIL	Pleomorphic high-grade squamous intraepithelial lesion
HPV	Human papillomavirus
SCC	Squamous cell carcinoma
ASC-H	Atypical squamous cells, cannot exclude high-grade squamous intraepithelial lesion
ASC-US	Atypical squamous cells of undetermined significance
NILM	Negative for intraepithelial lesion or malignancy
CIN	Cervical intraepithelial neoplasia
HR	High-risk
NA	Not available
BCD	Bizarre cell dysplasia
